# Altered Lipid Tumor Environment and Its Potential Effects on NKT Cell Function in Tumor Immunity

**DOI:** 10.3389/fimmu.2019.02187

**Published:** 2019-09-18

**Authors:** Shweta Tiwary, Jay A. Berzofsky, Masaki Terabe

**Affiliations:** ^1^Vaccine Branch, Center for Cancer Research, National Cancer Institute, NIH, Bethesda, MD, United States; ^2^Neuro-Oncology Branch, Center for Cancer Research, National Cancer Institute, NIH, Bethesda, MD, United States

**Keywords:** lipid metabolism, tumor immunity, natural killer T-cells, antigen presentation, dendritic cells

## Abstract

Natural killer T (NKT) cells are CD1d restricted T cells that mostly recognize lipid antigens. These cells share characteristics with both adaptive and innate immune cells and have multiple immunoregulatory roles. In a manner similar to innate immune cells, they respond quickly to stimuli and secrete large amounts of cytokines, amplifying and modulating the immune response. As T cells, they express T cell receptors (TCRs) and respond in an antigen-specific manner like conventional T cells. There are at least two subtypes of NKT cells, type I and type II, that differ in the nature of their TCR, either semi-invariant (type I) or diverse (type II). The two sub-types generally have opposing functions in tumor immunity, with type I promoting and type II suppressing tumor immunity, and they cross-regulate each other, forming an immunoregulatory axis. The tumor has multiple mechanisms by which it can evade immune-surveillance. One such mechanism involves alteration in tumor lipid repertoire and accumulation of lipids and fatty acids that favor tumor growth and evade anti-tumor immunity. Since NKT cells mostly recognize lipid antigens, an altered tumor lipid metabolic profile will also alter the repertoire of lipid antigens that can potentially affect their immune-modulatory function. In this review, we will explore the effects of alterations in the lipid metabolites on tumor growth, antigen cross-presentation, and overall effect on anti-tumor immunity, especially in the context of NKT cells.

## Introduction

Natural killer T cells (NKT cells) are a specialized subset of T-lymphocytes that share characteristics of both the innate and adaptive immune system. By definition, NKT cells are cells that recognize mostly lipid antigens presented by a non-classical class I MHC molecule, CD1d (1). CD1d is a member of the CD1 family, which are involved in presentation of a variety of both endogenous and exogenous lipid antigens to T-lymphocytes (2). NKT cells respond quickly and produce copious amounts of cytokines, further amplifying the immune response, while at the same time acting in an antigen specific manner. They are further categorized into two broad subsets based on their TCR repertoire. Type I NKT cells express a TCRα chain with limited diversity and therefore are referred to as semi-invariant NKT cells or invariant NKT cells (iNKT). The TCRα chain expressed by type I NKT consists of Vα14Jα18 in mice and Vα24Jα18 in humans, which preferentially pairs with Vβ8, Vβ7, Vβ2 in the former, and Vβ11 in the later (3–5). A marine sponge-derived lipid, α-GalCer (α-galactosylceramide) bound to CD1d, is a prototype ligand that binds to and activates virtually all type I NKT cells. In mice, type I NKTs are mostly CD4 single positive and CD4/CD8 double negative cells, whereas in humans these are CD4 or CD8 single positive as well as double negative cells (6). Type II NKT cells are a distinct CD1d restricted NKT population that does not react to α-GalCer. These cells express a more diverse TCR repertoire. A subset of type II NKT cells that reacts to sulfatide, a self-glycolipid, was the very first subtype to be identified by a specific ligand (7). Although type II NKTs can recognize a variety of lipids presented by CD1d, to date, sulfatide reactive type II NKT cells remain one of the best-described subsets (8). Type II NKT cells appear to be the predominant population in humans (9), but due to the lack of a specific ligand and isolation techniques, they have been difficult to study (10). Although NKT cells recognize lipid antigens, they can recognize hydrophobic peptides in addition to lipids as well, which is beyond the scope of this review and is reviewed elsewhere (11–14). Both Type I and II NKT cells modulate the immune response during tumor development and progression. Although highly contextual, in general, type I NKT cells are shown to have enhanced anti-tumor immune response whereas type II NKT cells generally act in an opposing manner (5, 15–18). However, in some mouse tumor models, type I NKT cells also have been shown to be suppressive of tumor immunity (18–22).

NKT cells recognize a diverse repertoire of both endogenous and exogenous lipids (2, 23). Most information on NKT lipid antigenic repertoire has come from mouse studies. Unlike humans, mice express only CD1d among the CD1 gene family (24). The generic structure of a lipid antigen-loaded to the CD1d molecule consists of a polar headgroup (e.g., a galactose sugar) linked to hydrophobic side chains. The CD1d molecule has two hydrophobic pockets, the A′ and F′ pockets, into which the hydrophobic side chains fit, whereas the polar headgroup sits outside and interacts with the TCR on the NKT cell (13). The length of the hydrophobic side chain as well as structural modifications in both the side chain and the polar headgroup can affect the binding of the lipid antigen presented by CD1d to the TCR on NKT cells. This, in turn, can have a differential effect on their activation status and eventual immune responses (25, 26).

Studies have reported several lipids that bind to CD1d and can potentially be presented to NKT cells. Glycerophospholipids and sphingolipids are the two major lipid groups that bind to CD1d (27). Phosphatidylcholine (PC), phosphatidylethanolamine (PE), Phosphatidylserine (PS), phosphoinositoI, phosphatidylglycerol, and phosphatic acid are the various glycerophospholids that have been shown to bind to CD1d with variable affinities. Several self-lipid antigens stimulate both murine and human NKT cells (28) such as lysophosphatidylethanolamine, and lysophosphatidic acids. Some lipids stimulate type I over type II NKT cells and vice versa. In particular, lysosphingomyelin stimulate only human type I NKT cells. Lysophospahtidylcholine stimulate both type I and type II NKT cells in humans, however, its reactivity with type I NKT is weaker. Additionally, lysophosphatidylcholine also reacts with murine type II NKT cells (29).

There are thousands of lipids within a mammalian cell serving functions ranging from energy storage to structural integrity to signal transduction (30). Any change in the lipid repertoire can disrupt tissue homeostasis leading to cellular transformation, cell proliferation, and migration (31–33). In this review, we will discuss the effect of altered lipid composition on tumor growth, anti-tumor immunity both NKT cell dependent and NKT cell independent. Some of the mechanisms by which lipid changes can modulate NKT cell dependent immune functions, directly or indirectly, that will be discussed here are (1) alteration in the quality of lipid antigen repertoire that can be presented to NKT cells, (2) impaired antigen cross presentation by DCs either by affecting the antigen processing machinery or MHC and CD1d surface expression, (3) modified quality and quantity of lipid reactive NKT cells, and (4) homing of NKT cells to the tumor sites.

## Altered Lipid Metabolic Status and Effect on Tumor Growth

Lipids are integral components of the cellular membrane where they participate in lipid raft formation and impact signal transduction (34). Thus, lipids have both structural and functional roles in maintaining cellular homeostasis. Fatty acids (FA) and cholesterol are the building blocks of all lipids in the body and are synthesized *de novo* in specialized tissues from Acetyl CoA. Other than synthesis, FAs are also taken up by the cells from the surroundings such as circulation, nearby tissues, and diet. Short chain saturated FAs are further elongated and desaturated by a specific set of enzymes to generate mono and polyunsaturated fatty acids (31). The human body is unable to synthesize long-chain polyunsaturated fatty acids (PUFAs) called omega 3 (DHA, docosahexaenoic, and EPA, eicosapentaenoic acid) fatty acids and omega 6 (arachidonic acid) at a reasonable rate and therefore, supplementation is required through dietary sources (35, 36). Alteration in lipid repertoire, such as saturated vs. unsaturated lipids, can influence multiple cellular functions. To illustrate, an altered lipid repertoire can impact membrane fluidity, cell-cell interaction, as well as the membrane protein landscape, which in turn can affect the downstream signaling cascade (37, 38). There are several studies that have reported a metabolic reprograming favoring *de novo* synthesis of lipids in cancer (39, 40). Additionally, an association between increased uptake of saturated fatty acids and cancer development has been reported in multiple cancer types (41–44). Also, a diet high in polyunsaturated fatty acids, especially omega 3s, have been shown to be negatively associated with cancer development (45–47). Consistent with that, one recent study reported a significant loss of PUFA especially omega 3 in breast cancer brain metastasis, by downregulation of its specific receptor, Major Facilitator Superfamily Domain Containing 2a (MFSD2a) on tumor endothelium (48).

**Figure 1:**
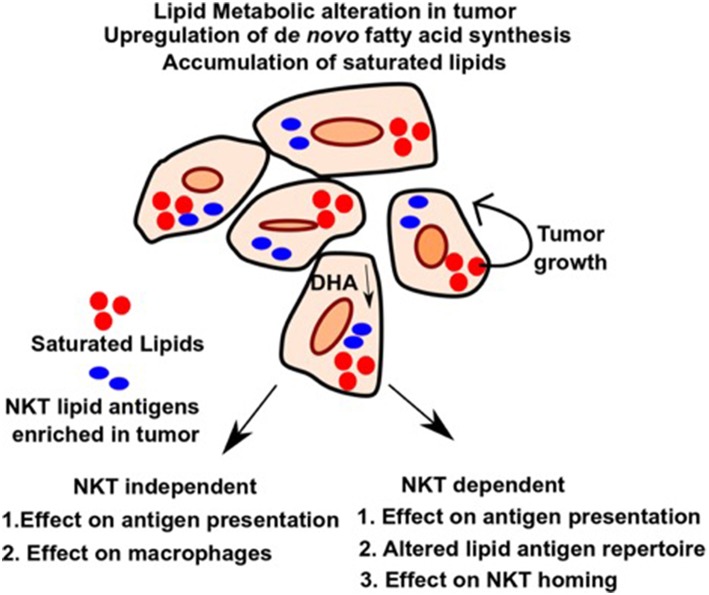
Alteration in lipid metabolism in tumor and potential effects on NKT independent and dependent immune function. Upregulation of *de novo* pathway and loss of tumor suppressive lipids such as DHA leads to differential accumulation of lipids in tumors, which favors tumor growth and provides energy sources and building blocks for bio-membranes. Alteration in lipid pool can affect immune response in an NKT independent or NKT dependent manner as outlined in the figure. It can lead to impaired macrophage function during inflammation and defective antigen presentation. Additionally, altered lipids can also serve directly as antigens for NKT cells and modulate their role in anti-tumor immunity. Homing of NKT cells can also be affected by altered lipids. Therefore, identifying the lipids as well as the pathways that lead to their upregulation and blocking it, can have potential therapeutic benefit in cancer.

Tumor cells have high metabolic flux. To sustain growth, they need a rapid and constant supply of FAs and lipids to generate bio-membrane, which is achieved by uptake of FAs from the surrounding tissues as well as upregulation of endogenous lipogenic pathways (49). Figure 1 outlines the effects of altered lipid metabolism on tumor growth as well as anti-tumor immunity. One pioneering study showed that tumor cells, in addition to uptake from the surrounding tissues, can also synthesize fatty acids *de novo* (39). Additionally, tumors can upregulate metabolic pathways leading to the accumulation of specific fatty acids and lipids that promote tumor growth and exclude those that suppress it. Consistent with that, various studies identified upregulation of several key lipid metabolic enzymes (such as ACC, Acetyl Co-A carboxylase, FASN, Fatty acid synthase, and ACLY, ATP-citrate lyase) under tumor conditions, and suppression of these enzymes involved in fatty acid synthesis has been shown to be preventive against tumor growth and metastasis (50–52). Additionally, sterol regulatory element-binding protein (SREBP), a master regulator of lipid biogenesis (53), is aberrantly upregulated in multiple cancer types and leads to upregulation of its target genes, promoting cancer growth (54). Furthermore, genetic or pharmacological inhibition of SREBP in pre-clinical studies, shows anti-tumorigenic effect by altering tumor specific lipid metabolism (55, 56).

## Effects of Altered Cellular Lipids on NKT Cell Independent Immune Responses

Lipid mediators are at the crux of both initiating an inflammatory response as well as resolving it (57–59). Therefore, metabolic deficiencies, pathogenic conditions, tumors, and dietary habits can cause an imbalance in the lipid metabolism that can skew the balance toward the accumulation of certain lipids over others, leading to aberrant immune activation.

### Effect of Altered Lipid Metabolism on Antigen Presentation

A high-fat diet that predominantly contains saturated fatty acids (SFAs) positively correlates with cancer development and progression (60–62). Although, both SFAs and PUFAs can have immunomodulatory effects under various pathological conditions (63), their effect on the immune system in the context of cancer development and progression is not well-understood. Many cancers accumulate SFAs by upregulating the *de novo* fatty acid synthesis pathway. These SFAs are preferentially taken up from the surrounding milieu. Additionally, tumors exclude PUFAs from their lipid pool. Alterations in the fatty acid pool of a cell can lead to gene expression changes as well as structural changes in the bio-membrane. Not much is known about the effect of altered lipid metabolism on lipid antigen presentation, recognition, and consequent activation of cytotoxic T cells (CTLs) and NKT cells, especially in cancer.

**Figure 2:**
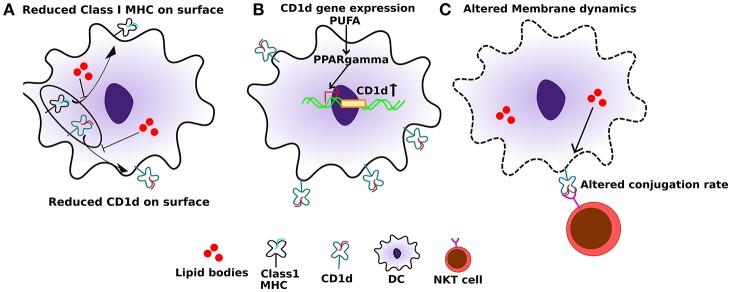
Effect of altered lipids on antigen presentation. **(A)** Accumulation of lipid bodies, mostly saturated lipids, negatively affects the localization of both class I MHC as well as CD1d. **(B)** Also, polyunsaturated fatty acids (PUFAs), especially DHA, can induce PPARγ levels in dendritic cells which in turn induce the expression of CD1d. **(C)** Lipid excess can also affect membrane dynamics, which in turn can interfere with CD1d:lipid antigen conjugation rate with TCRs on NKT cells, leading to sub-optimal NKT function.

Dendritic cells (DCs) are the professional antigen presenting cells in the body. Efficient antigen presentation by DCs results in enhanced activation and the cytotoxic response of CD8^+^ T cells. Several studies have shown that a high-fat diet, enriched in SFAs, can significantly impair the ability of DC's to activate naïve T cells. In addition to SFAs, PUFAs can also diminish the immunogenic function of DCs (64). APCs, when treated with high levels of palmitic acid (PA), express significantly reduced levels of class I MHC on their cell surface (Figure 2A). Additionally, this also leads to an impaired conjugation rate of APCs and lymphocytes (65) (Figure 2C). This effect is primarily due to altered membrane dynamics, and defects in membranes generated by high PA. Furthermore, co-treatment of oleic acid (a monosaturated fatty acid) with PA, sequesters PA into lipid droplets and negates its effect on cytoskeletal organization. This has important effects on antigen presentation and can thereby rescue the antigen presentation ability of APCs even when PA is present.

Other than treatment with exogenous fatty acids, endogenous fatty acids also affect DCs, both qualitatively and quantitatively. One study reported a significant reduction in the number of DCs as a result of blocking cell intrinsic fatty acid synthesis (66). However, their antigen presentation ability was not compromised. The study further reported a diminished maturation, yet an upregulated expression of TLRs on DCs upon inhibition of FA synthesis. Additionally, blocking FA synthesis led to increased production of inflammatory cytokines as well as enhanced antigen capture by the DCs. Taken together, these data suggest that an immune response elicited by DC-mediated antigen presentation, irrespective of peptide or lipid antigen, is highly contextual under physiological conditions and is dependent on the nature and levels of fatty acids.

Tumor cells can alter the DCs causing them to become dysfunctional and inefficient in antigen presentation (67). DCs can take up lipids from the tumor microenvironment, which can significantly affect their antigen presentation ability and hence immunogenicity (68). During growth tumors accumulate high levels of triglycerides (TAGs). DCs from a tumor-bearing mouse become significantly enriched for TAGs when compared to DCs from a naïve mouse. Further, this accumulation of lipids in the DCs from tumor-bearing mice is mainly by upregulation of scavenger receptor A in DCs. Additionally, high lipid content in DCs from tumor-bearing mice negatively affects the antigen processing machinery (69, 70). Also, the DCs in peripheral blood in persons with cancer show a lipid excess, and their numbers as well as their antigen presentation ability is significantly compromised (71). One hypothesis why DC vaccines or DC-based cancer therapies may not work is due to the accumulation of lipids when these cells are either in circulation or in the tumor microenvironment and a subsequent loss of antigenicity. If that turns out to be the case, then use of autologous monocytes to produce autologous DCs *ex vivo*, pulsing or transducing them with antigen, and maturing the DCs *in vitro* could produce tumor-targeted DC vaccines that evade this suppressive mechanism in the tumor microenvironment. Such a strategy is already being applied to avoid other immunosuppressive effects of tumors on DC maturation (72, 73). Interestingly, the defects of DC function induced by high lipid content seems to be reversed by reducing the lipid levels, thereby restoring their antigen presentation function and enhanced efficacy of DC-based cancer vaccines (69). Recently, one study reported defective antigen cross-presentation by tumor-associated DCs due to the accumulation of lipid bodies in the DCs containing oxidatively truncated lipids. The defect in the cross-presentation was due to impaired trafficking of MHC class I molecules to the cell surface (74). Another recent study reported an impaired antigen presentation of peripheral blood DCs in late-stage lung cancer patients due to high levels of TAGs (71). Together, these data suggest that an altered lipid environment in the tumor environment can directly affect DC function, both at the tumor site and peripherally.

### Effects of Altered Lipids on Macrophages

**Figure 3:**
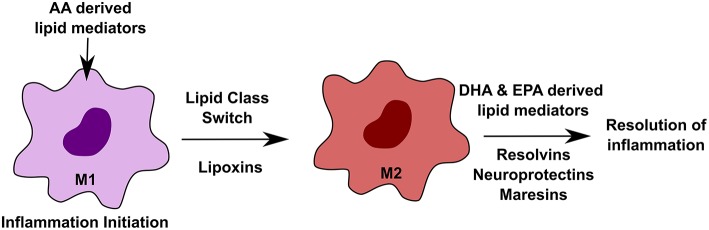
Effect of altered lipids on macrophages. Macrophages play important roles during inflammation in a highly orchestrated manner. During initiation of inflammation, arachidonic acid (AA)-derived lipid mediators, such as prostaglandins and leukotrienes, are required. M1 to M2 transition is mediated by lipoxins, which are also derived from AA. The resolution state of inflammation requires docosahexaenoic acid (DHA) and eicosapentaenoic acid (EPA) derived lipid mediators such as resolvins, neuroprotectins, and maresins.

Macrophages are diverse cell population found in every tissue (75). Tissue-specific environmental cues define their characteristics (76, 77). During inflammatory conditions, macrophages play distinct roles in an orchestrated manner, where initiation state is marked by the M1 phase, whereas, the M2 phase defines the beginning of the resolution, re-epithelialization and return to the homeostatic stage (78). Both M1 and M2 phenotypes of macrophages are dependent on specialized lipid mediators. A lipid class switch from pro-inflammatory AA (arachidonic acid) derived lipid mediators to an anti-inflammatory, DHA (docosahexaenoic acid) and EPA derived lipid mediators is important to push the macrophages to the resolution state, thereby inhibiting inflammation and re-establish homeostasis (58). Figure 3 outlines the effect of different lipids on macrophage function in inflammation. Tumor-associated macrophages (TAMs) play roles in promoting tumor growth. One study recently reported that debris generated by chemotherapy in tumors can stimulate TAMs to secrete pro-inflammatory cytokines thereby facilitating tumor growth. This effect was reversed by resolvins, which are a class of pro-resolving lipid mediators generated by DHA, thereby stimulating debris clearance by macrophages and suppression of tumor promoting inflammation (79).

## Effects of Altered Lipids on NKT Cell Functions

Alteration in cellular lipids can directly influence NKT cell function via affecting antigen cross presentation by DCs, altered lipid antigen repertoire leading to different CD1d:lipid complexes that are presented to NKT cells, and modulating expression of CD1d. Here, we will outline the effect of altered lipid repertoire in metabolic defects and cancer on NKT cell function as well as CD1d expression on DCs. Since antigen cross presentation can also influence NKT independent immune responses, we will cover that in a separate section.

### Effects of Metabolic Disorders on NKT Cell Development

Lipids are essential for development of NKT cells (80). Mice deficient in a lysosomal enzyme β-galactosidase (β-Gal) or lysosomal lipid transfer enzyme Niemann Pick C (NPC) 2 have reduced numbers of lipid-reactive type I NKT cells (81). This is largely due to defective CD1d antigen presentation and impaired thymic selection of type I NKT cells. Even though the number of NKT cells is reduced, there are still residual NKT cells with differential TCR Vβ usage and CD4 expression in both β-Gal^−/−^ and NPC2^−/−^ mice. This effect is due to the accumulation of different lipids leading to altered CD1d: lipid antigen complex formation. This in turn gives rise to NKT cells with different functional subsets where a significant decrease in Vβ8.2/Vβ7 ratio in β-Gal^−/−^ but not in NPC2^−/−^ was observed, in contrast to an increased ratio of CD4^−^/CD4^+^ in NPC2^−/−^ but not in β-Gal^−/−^ mice was observed. This suggests a direct effect of the type of lipid antigen presented on both quality and quantity of NKT cells. Several other mouse models of the lysosomal storage disease (Tay-Sachs, GM1 gangliosidosis, Fabry, NCP1) also show a reduced number of type I NKT cells, not due to defective CD1d presentation or lack of APCs, but due to impaired loading of lipid antigen on to the CD1d molecule (82). In addition to the decreased number, some lysosomal mouse models also show a defective function of type I NKT cells (82). Interestingly, in human patients with lysosomal storage disease, harboring NPC1 mutations, there does not appear to be any change in the number of type I NKT cells. Additionally, APCs from the patients can present lipid antigens to type I NKT cells efficiently (83). Although the quantity remains unchanged, the effect on the quality of type I NKT cells in response to altered lipids in lysosomal storage disease (84) is not known in humans.

### Effect of Altered Lipids on CD1d Antigen Presentation

DCs are professional APC that carry antigens from local tissues to the draining lymph nodes and are necessary to prime T cells including NKT cells. For the NKT cell priming, the expression level of CD1d is critical. One study reported increased expression of CD1d on human keratinocytes undergoing terminal differentiation upon increased cellular ceramide synthesis as well as exogenous ceramide application (85). Under physiological conditions, one study showed that peroxisome proliferator-activated receptor γ (PPARγ) upregulates CD1d in monocyte-derived DCs at the transcriptional level (86) (Figure 2B). Moreover, PPARγ mediated upregulation of CD1d is via activation of the retinoic acid pathway. PPARγ also enhances internalization activity and effective lipid antigen presentation to iNKT cells, leading to their activation and expansion, when α-GalCer is present (87). Interestingly, DHA-derived lipid mediators act as potential PPARγ agonists (88). Also, DHA has been reported to generate a tumor suppressive effect via PPARγ (89, 90). Consistent with that DHA can specifically upregulate PPARγ expression and levels of its target genes in DCs, and this upregulation is reversed by blocking PPARγ activity (91). However, DHA and lipid mediators derived from it are missing from the tumor environment (48). Several studies report an anti-tumor effect of DHA. DHA dietary supplementation, as well as its use as an adjuvant, has been shown to improve disease outcome in cancer patients (92). Additionally, PPARγ functions as a tumor suppressor and its expression is lost in many cancers (93). We can hypothesize that accumulation of tumor specific lipids in the tumor microenvironment can affect the expression of CD1d on both tumor cells and DCs, thereby suppressing their immunogenicity and facilitating eventual immune evasion. Immunogenic cell death as a result of intratumoral treatment of tumors with anti-cancer agents can lead to release of tumor-specific antigens, which then can activate T-cell mediated immunity and confer long term immunologic memory against tumor (94). The use of EPA/DHA alone or in combination with various chemotherapeutic agents has shown anti-tumor effects, mostly via apoptosis (92). We propose that co-treatment of tumors with EPA/DHA and intratumoral anti-cancer agents may provide a novel effective immunotherapy by mediating presentation of tumor antigens to T-cells and induction of long term anti-cancer immunity.

### Effects of Altered Lipids on NKT Cell Function in Inflammation and Cancer

Non-alcoholic fatty liver (NAFLD) is considered as a pre-malignant stage in the liver. One study in an obese mouse model for NAFLD reported a reduction in the number of hepatic NKT cells, as a result of activation-induced death of NKT cells by activated Kupffer cells due to lipid excess (95). Additionally, lipid excess in high fat diet (HFD)-induced obese mice activates type I NKT cells and skews the balance toward a pro-inflammatory cytokine environment. Further, lipid excess also causes obesity-induced insulin resistance and hepatic steatosis in an NKT dependent manner and can be reversed by deficiency of either type I NKT cells or CD1d (96) Another study reported a role of type II NKT cells in HFD induced obesity in mice (97). The study reported minimal weight gain, reduced inflammation, hepatic steatosis and insulin resistance in CD1d^−/−^ mice compared to Ja18^−/−^ mice. In addition to that, a direct role of CD1d mediated presentation of endogenous lipid antigens to activate NKT cells in mice fed with HFD was shown (98). Moreover, deletion of CD1d in adipocytes led to decreased weight gain and higher insulin sensitivity in mice. In a contrasting study, type I NKT cells were reported to suppress diet induced obesity and development of type II diabetes. The study further showed an increased infiltration of pro-inflammatory macrophages and decreased type I NKT in adipocytes during development of obesity. Moreover, an adoptive transfer of iNKT into Jα18^−/−^ obese mice or α-GalCer treatment of WT mice abrogated obesity induced disorders (99). Yet another study, reported no difference in weight gain, insulin sensitivity, inflammation and liver steatosis between CD1d^−/−^ vs. WT mice when fed with HFD (100). In context of hepatocellular carcinoma (HCC) as a result of NAFLD, one study reported no significant change in the NKT cell number as a consequence of increased lipid content in the liver in a transgenic mouse model (101). Another study identified a subset of NKT cells reactive to lysoPC lipid species in myeloma patients (102). In Gaucher disease (GD), another pathology caused by a lipid metabolic defect, it was shown that accumulation of β-glucocyceramide (β-GL1-22) and glucosylsphingosine (LGL1) led to induction of a different subset of type II NKT cell in both mice and humans (103). This specific subset of type II NKT cells leads to aberrant activation of humoral immunity and increased risk of B-cell malignancy.

Ceramides are released when cancer cells are exposed to chemotherapeutics or ionizing radiation leading to apoptotic death of tumor cells (104, 105). As ceramide is a major species of lipid that can be presented by CD1d to be recognized by NKT cells, the activation of NKT cells by ceramides released from treated tumors likely modulates the anti-tumor immune response. Interestingly, in the 4T1 pre-clinical tumor model, radiotherapy in mice deficient in type I NKT cells significantly enhanced tumor regression compared to WT mice with intact type I NKT cells (106). Additionally, administration of α-GalCer, NKT cell agonist that induces strong anti-tumor immunity, did not enhance the response to radiotherapy in WT mice, suggesting a potential immunosuppressive role of type I NKT cells that were exposed to tumor-derived lipids.

Gangliosides are yet another sialic acid-containing diverse group of glycosphingolipids that bind to and activate a subset of NKT cells (107, 108). Any alteration in lipid repertoire can also lead to altered ganglioside milieu. In regard to that, gangliosides disialoganglioside 2 (GD2) and disialoganglioside 3(GD3) have been reported to be overexpressed in cancer and shown to regulate tumor growth and metastasis (109). Mice immunized with melanoma cells expressing GD3 were found to have GD3 reactive NKT cells that were shown to be CD1d restricted (110). Additionally, coimmunization of GD3 loaded APCs along with GM3 loaded APCs suppressed the type I NKT cell function (108). GM3 also suppressed IL-4 production but not IFN-γ by type I NKT cells in response to α-GalCer. Also, GM3 is expressed in several malignancies and targeting it by specific antibody has anti-tumorigenic activity (111–113). In an ovarian cancer model, GD3 was shown to be enriched in tumor microenvironment and inhibit NKT cell activation. Also, GD3 abrogated a α-GalCer mediated NKT cell activation *in vivo* and *in vitro* by competing for the binding to CD1d (114). Furthermore, increased VEGF levels in tumor enhances GD3 levels in ovarian cancer (115). CD1d expressing APCs treated with GD3 significantly suppress NKT cell activation, suggesting a direct role of GD3 as a lipid antigen enriched in tumor in suppressing anti-tumor immunity in an ovarian cancer model through presentation by CD1d to NKT cells. Additionally, both GD3 and GM3 were recently reported to be present in TLR9 stimulated DCs (116) and synthetic versions of β-linked GM3 and GD3 were able to activate type I NKT in mice, both *in vivo* and *in vitro* in a CD1d dependent manner. Taken together, an altered lipid environment in the inflammatory conditions and tumor microenvironment can potentially affect NKT cell function and fine tune the immune response. Understanding the biology behind this can open up several therapeutic avenues such as therapeutically targeting synthesis of tumor promoting (e.g., GD3) lipids and/or using tumor inhibitory lipids (e.g., DHA) as adjuvants to enhance anti-tumor immunity.

## Effect of Lipids on Homing of NKT Cells

Localization of an immune cell to the site of injury is critical for resolution of inflammation and tissue homeostasis. In cancer, there are very limited studies that report localizing of NKT cells to the tumor site. CCR2 (expressed by NKT cells) and CCL2 (expressed by a subset of MYCN non-amplified neuroblastoma cells) mediated homing of NKT cells to neuroblastoma was shown in subset of neuroblastoma patients. Also, the survival of patients with NKT cell infiltration was significantly longer than that of patients without infiltration (117). In a follow-up study, it was demonstrated that MYCN repressed the expression of CCL2, thereby preventing homing of NKT cells to the tumor site in both mouse models and human patients (118). Interestingly, MYCN inhibition resulted in reduced tumor growth and improved survival in a transgenic mouse model. At the same time, there was an accumulation of lipid droplets in neuroblastoma cells which were treated with MYCN-inhibitors, suggesting a potential role for lipid metabolites involved in tumor regression (119). Not much is known about the nature of the lipids and mechanisms by which they may affect the recruitment of NKT cells to tumor site, which remain open questions.

One of the early studies reported a role of leukocyte function associated antigen-1 (LFA-1) on accumulation of NKT cells in the liver and LFA-1 deficient mice were shown to have significantly fewer NKT cells. (120). Also, LFA-1-intercellular adhesion molecule 1 (ICAM1) interaction was shown to be critical for tissue resident NKT cells in mice, such that blocking of either LFA-1 or ICAM1 led to a rapid release of NKT cells in circulation, in a parabiotic mouse study. Furthermore, this LFA-1-ICAM1 mediated tissue homing of NKT cells was shown to be dependent on the transcription factor promyelocytic leukemia zinc finger (PLZF) (121). Yet another study revealed the role of a chemokine receptor CXCR6 expressed on the NKT cell surface, and its specific receptor, CXCL16 (a transmembrane chemokine which is expressed on liver, lung and spleen cells), in homing of CXCR6 expressing NKT cells to the liver (122). This pathway is also lipid-dependent because the gut microbiome's metabolism of lipid bile acids affects the induction of CXCL16 and thus NKT cell homing to the liver and ability to control liver cancer (123).

## Conclusions

To date, most immune therapy treatment regimens in cancer focus on peptide-antigen-recognizing conventional T cells. However, lipid-reactive NKT cells have emerged as one of the major immune-modulators in tumor immunity, in pre-clinical mouse models. Although contextual, it is generally acceptable that type I NKT cells exert anti-tumorigenic effect whereas type II NKT cells have an opposite effect. Notwithstanding that both type I and II NKT cells constitute a small percentage of lymphocytes as compared to the conventional T cells, both NKT cell types mediate substantial immunomodulatory effects. Therefore, a deeper understanding of their differential regulation under normal and tumor conditions could unravel novel therapeutic nodes that can prove beneficial for anti-tumor immune therapy. Deregulated lipid metabolism is reported in several cancers. Unlike functional studies of DNA and proteins, knowledge of both the structural and functional roles of lipids in the process of cellular transformation and tumor growth has lagged behind. Changes in lipids can have a global effect on immune response and can influence anti-tumor immunity in both NKT-dependent and NKT-independent manners. Functional studies focused on understanding these aspects of tumor immunity can provide some unique and clinically useful therapeutic interventions.

## Author Contributions

ST, MT, and JB wrote and edited the manuscript.

### Conflict of Interest Statement

The authors declare that the research was conducted in the absence of any commercial or financial relationships that could be construed as a potential conflict of interest.
